# From hormone replacement therapy to regenerative scaffolds: A review of current and novel primary hypothyroidism therapeutics

**DOI:** 10.3389/fendo.2022.997288

**Published:** 2022-10-05

**Authors:** Maria Heim, Ian J. Nixon, Elaine Emmerson, Anthony Callanan

**Affiliations:** ^1^ Institute for Bioengineering, School of Engineering, The University of Edinburgh, Edinburgh, United Kingdom; ^2^ Centre for Regenerative Medicine, Institute for Regeneration and Repair, The University of Edinburgh, Edinburgh, United Kingdom; ^3^ Department of ENT, Head and Neck Surgery, NHS Lothian, Edinburgh, United Kingdom

**Keywords:** thyroid, hypothyroidism, regenerative medicine, tissue engineering, levothyroxine

## Abstract

Primary hypothyroidism severely impacts the quality of life of patients through a decrease in the production of the thyroid hormones T3 and T4, leading to symptoms affecting cardiovascular, neurological, cognitive, and metabolic function. The incidence rate of primary hypothyroidism is expected to increase in the near future, partially due to increasing survival of patients that have undergone radiotherapy for head and neck cancer, which induces this disease in over half of those treated. The current standard of care encompasses thyroid hormone replacement therapy, traditionally in the form of synthetic T4. However, there is mounting evidence that this is unable to restore thyroid hormone signaling in all tissues due to often persistent symptoms. Additional complications are also present in the form of dosage difficulties, extensive drug interactions and poor patience compliance. The alternative therapeutic approach employed in the past is combination therapy, which consists of administration of both T3 and T4, either synthetic or in the form of desiccated thyroid extract. Here, issues are present regarding the lack of regulation concerning formulation and lack of data regarding safety and efficacy of these treatment methods. Tissue engineering and regenerative medicine have been applied in conjunction with each other to restore function of various tissues. Recently, these techniques have been adapted for thyroid tissue, primarily through the fabrication of regenerative scaffolds. Those currently under investigation are composed of either biopolymers or native decellularized extracellular matrix (dECM) in conjunction with either primary thyrocytes or stem cells which have undergone directed thyroid differentiation. Multiple of these scaffolds have successfully restored an athyroid phenotype *in vivo*. However, further work is needed until clinical translation can be achieved. This is proposed in the form of exploration and combination of materials used to fabricate these scaffolds, the addition of peptides which can aid restoration of tissue homeostasis and additional *in vivo* experimentation providing data on safety and efficacy of these implants.

## 1 Introduction

Hypothyroidism, caused by the underproduction of thyroid hormone, is a debilitating condition which severely impacts quality of life (1). It presents with a wide range of symptoms, displayed in **Table 1**, most commonly including depression, fatigue, weight gain, dry skin, and bradycardia, amongst others (16, 36–39). Untreated, hypothyroidism can result in a life-threatening myxedema coma (40). The prevalence of hypothyroidism is thought to be around 9.3% in women and 1.3% in men, but it is related to iodine intake and therefore varies widely in between countries (41). Additionally, the incidence of primary hypothyroidism has been suggested to increase with age; and specifically, a Danish study has indicated this increase to be around eightfold (42).

**Table 1:** Signs & Symptoms of Hypothyroidism and their impact on quality of life: This table details the signs and symptoms of hypothyroidism, along with the incidence rate and perceived impact on the quality of life of patients.

Reference	Author	Symptom	Incidence rate	Impact on quality of life
(2)	Freinkel, 1972	Alopecia/Hair loss	33.30%	Low
(3)	Iorizzo, 2004	Brittle nails	90%	Low
(4)	Zhang, 2018	Cold Intolerance	33.33%	Low
(5)	Lang, 1978	Dry skin	90%	Low
(6)	Junuzovic-Zunic, 2019	Dysphonia	2-98%	Low
(7)	Bates, 2018	Loss of libido	33-60%(F), 8-52% (M)	Low
(8)	Kumar, 2012	Madarosis/Hertoghe sign	-	Low
(9)	Sadek, 2017	Shortness of breath	-	Low
(10)	Krishnamurthy, 2018	Woltman sign	12.50%	Low
(11)	Periasamy, 2016	Anaemia	20-80%	Medium
(12)	Logantha, 2016	Bradycardia	-	Medium
(13)	Karne, 2016	Carpal tunnel syndrome	16.70%	Medium
(14)	Taddei, 2003	Diastolic hypertension	30%	Medium
(15)	Gunsar, 2003	Dyspepsia	-	Medium
(16)	Louwerens, 2012	Fatigue	50%	Medium
(17)	Nedrebo, 1998	Hyperhomocysteinemia	-	Medium
(18)	Nagasaki, 2007	Increased C-reactive protein	-	Medium
(19)	Duntas, 2004	Increased serum cholesterol	-	Medium
(20)	Weeks, 2000	Menorrhagia	36.00%	Medium
(21)	Fariduddin, 2021	Myopathy	80%	Medium
(22)	Gupta, 2016	Peripheral and Central Neuropathy	-	Medium
(23)	Hoogwerf, 1984	Weight gain	60%	Medium
(24)	Ji, 2006	Abdominal Ascites	4%	High
(25)	Duret, 1971	Constipation	60-80%	High
(26)	Yaylali, 2009	Decreased gastrointestinal motility	-	High
(27)	Bode, 2021	Depression	-	High
(28)	Kiss, 1994	Impaired cardiac contractility & diastolic function	-	High
(29)	Samuels, 2014	Impaired memory	-	High
(30)	Danzi, 2003	Increased systemic vascular resistance	-	High
(31)	Wiersinga, 2018	Myxoedema coma	-	High
(32)	Chahine, 2019	Pericardial effusion	3-37%	High
(33)	Koyyada, 2020	Primary Infertility	37.10%	High
(34)	Altay, 2005	Rhabdomyolysis	-	High
(35)	Tsai, 2020	Sensorineural hearing loss	-	High

‘-’ signifies that no conclusive data could be found for the incidence rate of a sign or symptom during this review process. ‘M’ signifies incidence rate for males, ‘F’ signifies incidence rates for females. Impact on the quality of life by these symptoms was categorized with the following criteria: Low - minor discomfort or cosmetic changes, targeted symptomatic treatment seldom required. Medium – increased discomfort, impact on activities of daily living, targeted symptomatic treatment may be required. High – acute discomfort, significant disruption of activities of daily living, increased risk of medical emergency or frequently required targeted symptomatic treatment.

In accordance with its aetiology, hypothyroidism can be divided into three subtypes: primary; central, comprised of secondary and tertiary; and peripheral (42). Primary hypothyroidism, which accounts for 99% of all hypothyroidism cases, arises due to an inability of the thyroid to produce the thyroid hormones triiodothyronine (T3) and thyroxine (T4) (41). This can be attributed to a variety of reasons including medication and iodine deficiency (43, 44). However, the majority of causes in developed countries are instead due to direct damage of the thyroid gland through processes like inflammation, autoimmune disease, or radiation (45–47).

For example, the most common clinically late effect of radiation, when applied at a therapeutic dose (30-70Gy) within the cervical region, is primary hypothyroidism (46, 48). The overall incidence of hypothyroidism following irradiation in the head or neck region varies widely between studies, ranging up to 92%, but most frequently lays between 20-60% (49). The exact pathological mechanism through which radiation induces primary hypothyroidism remains to be eluded but radiation is known to directly cause gland damage through induction of apoptosis, fibrosis of the gland capsule, atherosclerosis, thyroiditis, thyroglobulin antibodies (50–54).

Considering the direct correlation between primary hypothyroidism and radiotherapy for head and neck cancer, as well as the observed significant increase in survival rates of cancer, rising from 24% to 50% over the last 40 years, and a latency period of up to 27 years for radiation-induced hypothyroidism, we can expect to see a significant increase in the incidence of primary hypothyroidism (55). In addition, the correlation between age and an increased prevalence of hypothyroidism, in a steadily ageing population, will contribute to an overall higher incidence of hypothyroidism in the near future.

Here, we will review current primary hypothyroidism treatment methods and novel therapeutic approaches. The novel approaches currently under investigation employ regenerative medicine and tissue engineering strategies, primarily focusing on regenerative scaffolds. This type of therapeutic may be applicable against various conditions affecting the thyroid gland. Thus, we have taken care to specifically select therapeutics produced with a purpose for application against primary hypothyroidism and qualities needed to treat this condition.

## 2 Treatment of primary hypothyroidism

### 2.1 Current approaches

Currently, there is no distinction between the treatments associated with primary hypothyroidism based on causality. As with the majority of endocrinological conditions induced by a lack of hormones, primary hypothyroidism is treated through hormone replacement therapy (HRT) (56). This is required to be taken for life if the disease cannot be treated through different methods.

#### 2.1.1 Desiccated thyroid extract

The first regimen of thyroid HRT was described in 1891 by Murray and included the subcutaneous injection of sheep thyroid extract (57). Soon after, it was shown that the oral administration of thyroid extract proved to be just as effective (58). Desiccated thyroid extract (DTE) is thyroid gland tissue which has been lyophilized; it contains both T4 and T3 (59).

The majority of DTE used today is generated from pigs. However, there is an extensive dispute around the usage of DTE to treat any form of hypothyroidism, following the introduction of synthetic hormones (56). An argument against the use of pig DTE is the T4:T3 ratio, commonly around 4:1, in contrast to the human physiological ratio of 14:1 (60, 61). The treatment may therefore lead to a supraphysiological concentration of T3, which can lead to severe adverse effects such as those associated with thyrotoxicosis, including palpitations, heat intolerance and in severe circumstance loss of consciousness (62). Long-term, double-blind studies regarding the safety of DTE use for hypothyroidism are also lacking. Additionally, the contents of DTE preparations, excluding hormone concentrations, remain unregulated in many parts of the world (63). The use of DTE preparations is not currently approved by the Food and Drug Administration (FDA). However, as DTE was introduced into the market before FDA approval was implemented (“grandfathered in”), this is not a legal obligation and the contents and quality of DTE remains unregulated by authorities. Furthermore, clinical studies did not find a difference between the effectiveness of DTE and alternate HRTs, but most patients preferred DTE due to improvement of subjective symptoms and adverse effects (64, 65). This evidence highlights the need for further research regarding the use of DTE for hypothyroidism treatment.

#### 2.1.2 Synthetic T4 monotherapy

T4 crystals were originally purified by Kendall in 1914 and became commercially available soon after (66). 12 years later, Harrington and Barger were able to delineate the structure of T4 and synthetic T4 was available for clinical use by the 1930s (67). However, it was not commonly used until almost 20 years later, likely due to its high cost, when it was marketed similar to a completely new drug (68, 69).

Today, levothyroxine (L-T4) is the treatment of choice for primary hyperthyroidism (70, 71). Whilst the safety and effectiveness of L-T4 are well established, there are multiple drawbacks and issues associated with its therapeutic use. There is no one-size-fits-all approach to treatment with L-T4; monitoring of serum thyroid stimulating hormone (TSH) levels, which regulates thyroid hormone secretion, must be undertaken for the entire duration of therapy. It is recommended that TSH levels are measured upon starting treatment and following every dose change, as well as yearly following stabilization of the L-T4 dose (71). If too little L-T4 is administered, serum TSH levels remain high and symptoms of hypothyroidism persist, whilst if the dosage is too high, serum TSH levels may decrease excessively, and symptoms of thyrotoxicosis may be encountered. This includes parlous cardiac issues such as tachycardia, arrhythmias, palpitations (72). Overtreatment with L-T4, which leads to TSH levels below 0.1 mlU/L, has also been shown to be associated with adverse skeletal health, in the form of bone mineral density loss increasing risk for fractures and osteoporosis, especially in elderly patients (73, 74). Additionally, due to the positive inotropic and chronotropic effects of thyroid hormone on the heart, patients with pre-existing ischemic heart disease will need to be started on a low dose of L-T4, which is gradually titrated upwards (75, 76). Cardiac medication will need to be adjusted in some of these patients so that the adequate dose of L-T4 needed to maintain a euthyroid state can be tolerated (75).

Furthermore, several medications, supplements and foods can interfere with the absorption, metabolism, and efficacy of L-T4. A comprehensive list of common drugs and minerals which fall into the category of interacting with L-T4, but do not induce hypothyroidism by themselves, can be seen in **Table 2**. Estrogen and methadone increase the bound fraction of T4 indirectly through raising circulating levels of thyroxine-binding globulin (TBG), leading to a higher treatment dose of L-T4 required to achieve satisfactory thyroid hormone levels (79, 80). Glucocorticoids and anabolic steroids have the opposite effect, decreasing thyroid binding globulin concentrating, often requiring a reduction of the L-T4 dose (84, 85). Many antacids and foods or dietary supplements rich in iron, calcium, and magnesium, as well as fiber and caffeine decrease the gastric absorption of L-T4 (91, 94, 95). Multiple seizure medications and drugs with agonistic β-adrenoreceptor activities, are known to increase the clearance of L-T4, as they act as enzyme inducers, directly altering thyroid hormone metabolism (100, 101, 106, 111, 112). Lastly, some drugs alter the secretion of thyroid stimulating hormone, also leading to an increased dose of L-T4 being required (108, 110, 113). In some cases, instead of a therapeutic L-T4 dose increase, the interacting drugs or foods may also be taken 4 hours apart to minimize any adverse events (71).

**Table 2:** Overview of interactions with levothyroxine (LT4): Compounds which have been recorded to interact with LT-4 and their effect on LT-4 metabolism, distribution and absorption are listed.

Type of Interaction	Compound	Drug class	Possible action required	Author	Reference
Alters THST - ↑ TBG concentration	Capecitabine	Thymidylate synthase inhibitor	↑ L-T4 dose	Narula, 2004	(77)
Alters THST - ↑ TBG concentration	Clofibrate	Antilipidemic agent	↑ L-T4 dose	Christ, 1990	(78)
Alters THST - ↑ TBG concentration	Diamorphine	Opioid receptor agonist	↑ L-T4 dose	Christ, 1990	(78)
Alters THST - ↑ TBG concentration	Estrogens	Hormones	↑ L-T4 dose	Mazer, 2004	(79)
Alters THST - ↑ TBG concentration	Fluorouracil	Pyrimidine analogue	↑ L-T4 dose	Christ, 1990	(78)
Alters THST - ↑ TBG concentration	Methadone	Opioid receptor agonist	↑ L-T4 dose	English, 1988	(80)
Alters THST - ↑ TBG concentration	Mitotane	Steroidogenesis inhibitor	↑ L-T4 dose	Blanchet, 2016	(81)
Alters THST - ↑ TBG concentration	Raloxifene	Selective oestrogen receptor modulator	↑ L-T4 dose	Sing, 2003	(82)
Alters THST - ↑ TBG concentration	Tamoxifen	Selective oestrogen receptor modulator	↑ L-T4 dose	Mamby, 2016	(83)
Alters THST - ↓ TBG concentration	Anabolic-androgenic steroids	Steroid hormones	↓ L-T4 dose	Deyssig, 1993	(84)
Alters THST - ↓ TBG concentration	Glucocorticoids	Steroid hormones	↓ L-T4 dose	Emerson, 1993	(85)
Alters THST - Displaces T4 from protein-binding site	Furosemide	Loop diuretic	↑ L-T4 dose	Stockigt, 1985	(86)
Alters THST - Displaces T4 from protein-binding site	Mefenamic acid	Non-steroidal anti-inflammatory drug	↑ L-T4 dose	Koizumi, 1984	(87)
Alters THST - Displaces T4 from protein-binding site	Salicylates	Non-steroidal anti-inflammatory drug	↑ L-T4 dose	Larsen, 1972	(98)
↓L-T4 absorption	Aluminium hydroxide	Gastric antacid	↑L-T4 dose	Liel, 1994	(89)
↓L-T4 absorption	Bile acid sequestrants	Hypolipidemic agents	↑L-T4 dose	Northcutt, 1969	(90)
↓L-T4 absorption	Caffeine	Central nervous system stimulant	↑L-T4 dose	Benvenga, 2008	(91)
↓L-T4 absorption	Calcium carbonate	Gastric antacid	↑L-T4 dose	Singh, 2001	(92)
↓L-T4 absorption	Chromium Picolinate	Insulin receptor modulator	↑L-T4 dose	John-Kalarickal, 2007	(93)
↓L-T4 absorption	Ferrous sulphate	Dietary supplement	↑L-T4 dose	Irving, 2015	(94)
↓L-T4 absorption	Fibre	Dietary supplement	↑L-T4 dose	Liel, 1996	(95)
↓L-T4 absorption	Lovastatin	HMG-CoA reductase inhibitor	↑L-T4 dose	Demke, 1989	(96)
↓L-T4 absorption	Magnesium carbonate	Gastric antacid/Laxative	↑L-T4 dose	Mersebach, 1999	(97)
↓L-T4 absorption	Magnesium hydroxide	Gastric antacid	↑L-T4 dose	Mersebach, 1999	(97)
↓L-T4 absorption	Proton pump inhibitors	Proton pump inhibitors	↑L-T4 dose	Irving, 2015	(94)
↓L-T4 absorption	Sevelamer	Phosphate binder	↑L-T4 dose	John-Kalarickal, 2007	(93)
↓L-T4 absorption	Soy protein	Dietary supplement	↑L-T4 dose	Bell, 2001	(98)
↓L-T4 absorption	Sucralfate	Gastric protectant	↑L-T4 dose	Sherman, 1994	(99)
Alters of THM - ↑ hepatic metabolism	Carbamazepine	Tricyclic anticonvulsant	↑ L-T4 dose	Aanderud, 1981	(100)
Alters of THM - ↑ hepatic metabolism	Phenytoin	Anticonvulsant	↑ L-T4 dose	Finucane, 1976	(101)
Alters of THM - ↑ hepatic metabolism	Rifampicin	Antibiotic	↑ L-T4 dose	Nolan, 1999	(102)
Alters of THM - ↑ hepatic metabolism	Ritonavir	Protease inhibitor	↑ L-T4 dose	Sahajpal, 2017	(103)
Alters THM - ↓ T4 5’-deiodinase activity	Amiodarone	Class III antiarrhythmic/β-adrenoreceptor antagonist	↑ L-T4 dose	Basaria, 2005	(104)
Alters THM - ↓ T4 5’-deiodinase activity	Dexamethasone	Corticosteroid	↑ L-T4 dose	DeGroot, 1976	(105)
Alters THM - ↓ T4 5’-deiodinase activity	Propranolol	β-adrenoreceptor antagonist	↑ L-T4 dose	Kristensen, 1977	(106)
Alters THM –Mechanism unknown	Sertraline	Selective serotonin reuptake inhibitor	↑ L-T4 dose	McCowen, 1997	(107)
↓ TSH secretion	Amphetamine	Central nervous system stimulant	↑ L-T4 dose	Hein, 1990	(108)
↓ TSH secretion	Bexarotene	Selective retinoid X receptor agonist	↑ L-T4 dose	Sherman, 2003	(109)
↓ TSH secretion	Bromocriptine	Dopamine receptor agonist	↑ L-T4 dose	Ishihara, 1985	(110)
↓ TSH secretion	Dobutamine	β-adrenoreceptor agonist	↑ L-T4 dose	Lee, 1999	(111)
↓ TSH secretion	Dopamine	Dopamine/β-adrenoreceptor agonist	↑ L-T4 dose	Scanlon, 1979	(112)
↓ TSH secretion	Octreotide	Somatostatin analogues	↑ L-T4 dose	Christensen, 1992	(113)
↓ TSH secretion	Somatropin	Growth hormone	↑ L-T4 dose	Porretti, 2002	(114)

Whilst individual of these interactions may be true for multiple compounds of a drug class, only those with studies conducted are present. The recommended action in all cases where the LT-4 dose could be increased is for the compound and LT-4 to be taken 4 hours apart. Alternatively, a decrease of the LT-4 dose may be required, as indicated. ‘↑’ signifies an increase. ‘↓’ signifies a decrease. ‘THST’ stands for thyroid hormone serum transport. ‘TBG’ stands for thyroxine-binding globulin. ‘THM’ stands for thyroid hormone metabolism. ‘TSH’ stands for thyroid stimulating hormone.

Most failings of L-T4 therapy can be attributed to poor patient compliance (115, 116). This is often due to the limitations and inconvenience imposed by the therapy such as avoidance of the above discussed medications, daily administration of the medication, and food intake prior to as well as 30-60 minutes after ingesting L-T4 (117). Non-compliance with the prescribed therapy can lead to adverse metabolic and cardiovascular effects, as well as psychological events such as paranoia and depression, further impairing quality of life (118). Whilst it has been proposed that weekly and intermittent administrations of L-T4 could prevent these issues, a dose sevenfold higher would be required. High doses of L-T4 may lead to a condition known as iatrogenic thyrotoxicosis factitia, when tissue is exposed to high levels of circulating thyroid hormone, causing the patient to experience symptoms of thyrotoxicosis such as palpitations, sweating and anxiety (72).

These symptoms of iatrogenic thyrotoxicosis were correlated with increased levels of plasma hydroperoxides, which act as markers of oxidative stress. Additionally, these symptoms were relieved using physiological modulators which expressed antioxidant activities (119, 120). Together, this evidence suggests that chronic use of L-T4 provokes a hypermetabolic state which leads to an increased mitochondrial production of reactive oxygen species (ROS). This excessive production of free radicals gives rise to oxidative stress; a harmful process which can negatively affect several cellular structures including membranes, lipids, proteins, lipoproteins, and DNA (121). The resulting consequences of oxidative stress have been associated with adverse consequences including the promotion of tumorigenesis, cardiovascular disease, neurological disease, renal disease, and chronic inflammatory diseases (122–127). Whilst there are other synthetic T4 alternatives available to L-T4, none of these circumvent the above discussed issues encountered with L-T4 therapy.

#### 2.1.3 Synthetic combination therapy

In contrast to T4, serum T3 was discovered in 1952 by Gross and Pitt-Rivers (128, 129). A therapy with the combination of ‘natural’ T3 and T4 was originally employed as treatment for hypothyroidism through the use of DTE. As the use of DTE started being questioned, mainly due to discontent with variable potency, mixtures of synthetic T3, commonly liothyronine (L-T3) and L-T4 were employed instead (130). However, the landmark discovery that T4 can be peripherally converted into T3 in thyroid-deficient patients led to the replacement of LT3:LT4 combination therapy with L-T4 monotherapy as a first-line therapy (131).

Nevertheless, a significant minority of 5-10% of patients experience persistent symptoms related to hypothyroidism despite treatment with L-T4 (71, 132). Multiple studies have shown that many of these patients do not achieve the physiological free T3:T4 ratio, despite presenting with TSH serum level within the normal range (133, 134). This suggests an impairment of hepatic or renal conversion of T4 to T3, which may account for the persistence of the symptoms. Additionally, studies show that cerebral, hepatic, and skeletal muscle tissue of rats treated with L-T4 exhibit markers of localized hypothyroidism (135). Together this evidence indicates that L-T4/L-T3 combination therapy may be preferential.

Several studies have indeed evaluated the effect of T3/T4 combination therapy and obtained mixed results. A meta-analysis involving 1216 patients in 11 randomized controlled trials indicated no significant improvement in hypothyroid-related symptoms between combination and monotherapy (136). Another meta-analysis showed that 48% of patients preferred combination therapy, despite no improvement of objective symptoms, whilst 25% preferred T4 monotherapy, and 27% had no preference (71). It has been indicated that the results of these studies may be divergent due to single nucleotide polymorphisms (SNP) in genes involved in the thyroid hormone axis. This especially includes Thr92Ala in type 2 deiodinase (*DIO2*), essential for the peripheral conversion of T4 into T3 and present in the brain, pituitary, skeletal muscle, heart, brown adipose tissue, and thyroid (60, 137, 138). Indeed, studies have indicated that this particular SNP is associated with a significantly enhanced response to combination therapy and impaired baseline psychological well-being when on T4 monotherapy (139, 140). Multiple other SNPs have been indicated in genes associated with the regulation of the thyroid hormone axis (141–143). This implies that personalized medicine, based on genotype, may be the most ideal way of deciding the type of thyroid HRT needed. Further trials defining the influence of SNPs on and the safety and efficacy of T3:T4 combination therapy will need to be conducted before this can be implemented. The cost effectiveness of precision medicine approaches like this, and the restructuring of implemented healthcare systems required to implement these for a disease as common as hypothyroidism, may add considerable hurdles (144).

## 3 Experimental bioengineering and regenerative medicine approaches

The idea of transplantation to replace the thyroid gland and recover normal function, was originally proposed in the late 1800s by Sir Victor Horsley, through the suggestion that grafting a portion of healthy thyroid tissue may be a viable treatment method for myxoedema – now known to result from advanced hypothyroidism (145). Whilst this treatment initially showed success, it did not last long and with the recognition of the issues associated with organ transplantations, further attempts at utilizing this treatment method quickly diminished. However, with the advances in stem cell biology, thyroid progenitor cells were identified, revitalizing the idea of cell transplantation to rescue thyroid function. The harvesting and culturing of thyroid progenitor cells has proven to be a challenging task, hence other approaches to obtaining transplantable thyroid cells have been pursued. These have mainly focused on the utilization of embryonic stem cells (ESCs) or induced pluripotent stem cells (iPSCs), but also on primary thyrocytes. A list of studies employing tissue engineering and regenerative medicine approaches as treatment for primary hypothyroidism is displayed in **Table 3**.

**Table 3:** Novel bioengineering and regenerative medicine approaches to produce treatments for hypothyroidism.

Reference	Author	Scaffold Material	Cells Type	Study	Method	Result
(146)	Antonica et al., 2012	Matrigel	Murine ESCs	*In vivo* (mice)	Recombinant tetracycline inducible NKX2.1 & PAX8 ESC lines generated, which were differentiated into embryoid bodies in hanging drops and into thyroid follicles in Matrigel-supported 3D culture with addition of rhTSH and Dox. The organoids were grafted under the renal capsule of hypothyroid female mice.	Overexpression of NKX2.1 & PAX8 induced differentiation of ESCs into FTCs. Cells showed iodide organification activity and formed follicular organoid structures *in vitro*. These rescued thyroid hormone plasma levels *in vivo.*
(147)	Arauchi et al., 2009	NA	Primary rat thyroid cells	*In vivo* (rats)	Thyroids were minced & incubated with collagenase type II and IV before culturing on temperature responsive PIPAAm dishes. Cell sheets were implanted subcutaneously into the gluteus after 1-week of culture.	Serum T3 and T4 significantly increased 1 week after transplant and were maintained for 4 weeks. Morphological analysis showed typical follicle organization, microvessel formation and the presence of follicle epithelial cells.
(148)	Bulanova et al., 2017	Collagen hydrogel	Primary murine thyroid cells & Allantoids	*In vivo* (mice)	Thyroid spheroids and allantoic spheroids were used as a source of thyrocytes and endothelial cells. These were printed in close association within a collagen hydrogel and implanted into mice.	Epithelial cells invaded and vascularized thyrocytes leading to the progressive formation of follicles. These were able to restore body temperature and serum T4 levels in mice.
(149)	Gabr et al., 2018	Decellularized porcine liver organoid	Wharton Jelly derived MSCs	*In vivo* (mice)	Thyroid islets, produced through addition of Activin A and TSH, were loaded onto decellularized porcine liver organoids supplemented with TSH and iodine, then implanted intraperitoneal or intramuscular into mice.	Organoid corrected the hypothyroid state and showed viability and function at both implantation sites. Intramuscular transplantation showed higher vascularity and iodine uptake.
(150)	Kurmann et al., 2015	Matrigel	Murine iPSCs	*In vitro*	Nkx2-1(GEP); Pax8(tdTomato trace) iPSCs were generated and differentiation directed towards thyroid with specific factors to induce Nkx2-1 expression. Cells were cultured 2D or in 3D Matrigel for organoid production.	Successfully matured thyroid progenitors derived from mouse iPSCs into thyroid follicular organoids which secrete T3, T4 and TSH.
(151)	Martin et al., 1993	Matrigel	Human follicular thyroid cells	*In vivo* (mice)	Organoids were constructed from thyrocytes, embedded into a basement membrane preparation, and transplanted into SCID mice.	Widespread neofollicle formation was observed after 4 weeks of transplant. Murine T4 levels were not altered but human Tg was secreted and increased in response to TSH stimulation.
(152)	Mastrogiacomo, et al., 2012	3.5-5% PLLA in anhydrous dichloromethane/amylene or 3% PCL in anhydrous tetrahydrofuran/BHT	Primary rat thyroid cells	*In vitro*	The biomaterial scaffolds were prepared on wet glass support and cells seeded, allowing 8 days for growth.	Both biomaterial sheets promoted survival, adhesion, and proliferation of primary thyroid elements. T3 and T4 secretion varied but were a higher volume than in monolayer cultures.
(153)	Pan et al., 2019	Decellularized thyroid gland (Male Lewis Rats)	FRTL-5 or Primary human follicular thyroid & parathyroid cells	*In vitro*	Thyroid gland decellularized with 1% SDS and recellularized perfusion seeding	Retained ECM and vascular network of native thyroid. Recellularized thyroid-maintained expression of Tg, thyroid peroxidase, and parathyroid hormone.
(154)	Risheng Ma, 2015	Gelatin/Matrigel	Human ESCs	*In vitro*	Human PAX8 and NKX2-1 were expressed in ESCs with pEZ-lentiviral vectors, followed by differentiation into thyroid cells directed by Activin A and TSH within Gelatin/Matrigel	Double transfected cells expressed thyroid specific genes and the differentiation approach induced thyroid follicle formation. TSH stimulation induced dose-dependent cAMP generation and radioiodine uptake.
(155)	Strusi, V., et al., 2012	Decellularized thyroid gland (Sprague-Dawley Male Rats)	Primary follicular thyroid or ABCG2+ thyroid stem/precursor (S/P) cells	*In vitro*	Thyroid matrix obtained by freezing/detergent/enzyme processing. Cells were expanded in a monolayer or 3D Matrigel culture before scaffold recellularization.	Thyroid architecture and SVS were maintained. S/P initiated follicle formation. Thyroid hormones were secreted for 7 days (minimum).
(156)	Strusi, V., et al., 2011	Decellularized thyroid gland (Sprague-Dawley Male Rats)	Primary rat thyroid cells	*In vitro*	Rat thyroids were decellularized through freezing/thawing and sequential washes with 0.02% trypsin/0.05% EDTA, 3% Triton-x100, 4% deoxycholic acid and 0.1% peracetic acid (1% P/S/Fungizone). Cells were seeded in the inner surface of the matrix.	Native 3D architecture and the thyroid SVS were retained. Thyroid-derived cells aggregate, form intracytoplasmic cavities up to follicular coating and undergo secretory de-differentiation.
(157)	Weng, et al., 2021	Decellularized thyroid gland (New Zealand White Rabbits)	Human follicular thyroid cells	*In vitro*	Thyroid gland decellularized with 1% SDS and recellularized with HFTCs	Maintained biomechanical properties, ECM, and cytokine composition of the native thyroid. Thyroid peroxidase secretion present. No cytotoxicity was induced.
(158)	Yang et al., 2016	Alginate-poly-l-ornithine-alginate (APA)	Primary porcine thyroid cells	*In vitro*	Porcine primary thyroid cells were obtained through dissociation and collagenase digestions. The cells were suspended in low-viscosity-high-mannuronic acid solution, and the inner alginate core was formed by a microfluidic device. The inner core was coated to form an APA multilayer following cross-linking in a calcium chloride solution	The porcine cells successfully formed thyroid follicles in the microcapsule and displayed viability and proliferation. T4 was released significantly higher in encapsulated cells than unencapsulated cells.

NKX2.1, NK2 Homebox 1; PAX8, Paired Box 8; ESC, embryonic stem cell; rhTSH, recombinant human thyroid stimulating hormone; FTC, follicular thyroid cell; NA, Not applicable; PIPAAm, Poly(N-isopropylacrylamide); MSC, mesenchymal stem cell; iPSC, induced pluripotent stem cell; SCID, severe combined immunodeficient; Tg= Thyroglobulin; PLL, Poly-L-lactic acid; PCL, Poly-Ꜫ-caprolactone; BHT, butylated hydroxytoluene; FRTL-5, Fischer Rat Thyroid low serum 5%; SDS, Sodium Dodecyl Sulphate; ECM, extracellular matrix; EDTA, ethylenediaminetetraacetic acid; SVS, stromal vascular scaffold; S/P, Stem/Precursor; P/S, Penicillin/Streptomycin; HFTC, human follicular thyroid cell.

### 3.1 Biopolymer scaffolds

The use of biopolymer scaffolds has received much attention within regenerative medicine and bioengineering. They have been applied for the purpose of restoration and reconstruction of organ function in various tissues (159–162). Scaffolds are constructed of three-dimensional porous, fibrous, or permeable biomaterials with the intent of permitting transport of liquids and gases, promoting cell interaction, viability, and extracellular matrix (ECM) deposition whilst eliciting minimal inflammatory or cytotoxic responses (163). These scaffolds do not only provide mechanical support, but also serve as delivery systems for bioactive molecules and templates for cell adhesion, acting as an initiator for tissue neogenesis (164).

One of the earliest instances of utilizing biopolymer scaffolding to rescue thyroid function perhaps includes the transplantation of thyroid organoids, produced through culture of human thyrocytes in Engelbreth-Holm-Swarm mouse sarcoma – commercially known as Matrigel^®^ –, into severe combined immunodeficiency (SCID) mice (151). Whilst this transplantation did not alter murine serum T4 levels, evidence of thyroid follicle reconstitution along with neovascularization was found. Furthermore, human thyroglobulin was detected in the serum, which increased upon human TSH stimulation, indicating functionality and TSH responsiveness of the transplanted organoids.

Subsequent experiments indicated the importance of the expression of transcription factors NK2 homebox 1 (NKX2-1, previously TTF-1) and paired box 8 (Pax8) in thyroid development as well as the specific thyroid gene expression program (165). Consequent overexpression of Pax8 and NKX2-1 proved to be sufficient to direct mouse ESC differentiation into thyrocyte-like cells (146). Remarkably, transient expression of these transcription factors was able to induce long-term thyroid cell development, negating the need for outside induction of these genes. These induced thyrocytes formed follicular aggregates when cultured in 3D Matrigel and stimulated with TSH, which demonstrated polarized characteristics consistent with natural thyroid follicles. Upon engraftment into athyroid mice, these follicles rescued serum thyroid hormone levels and promoted symptomatic recovery. A similar approach involving overexpression of Pax8 and NKX2-1 in human ESCs cultured in a Gelatine/Matrigel system produced functional thyroid follicles upon Activin A and TSH stimulation (154).

Alternatively, to primary cells, iPSCs, generated from human dermal fibroblasts, have successfully been used to produce T3 and T4 secreting thyroid progenitors in Matrigel (150). This was achieved in NKX2-1 haploinsufficient lines, through stimulation with Activin A, transient inhibition of transforming growth factor (TGF) β and bone-morphogenetic proteins (BMP) signalling, followed by stimulation with fibroblast growth factor (FGF) 2 and BMP4 and maturation in Matrigel.

Notably, the above-described approaches all employed Matrigel or Matrigel-combination gels as a scaffold. To date, only one study has been published using an alternative biomaterial with the aim of rescuing a hypothyroidism phenotype. This involved the use of either poly-L-lactic acid (PLLA) or poly-caprolactone (PCL) polymerized on a wet glass support and seeded with primary rat thyroid cells (152). Both biomaterials increased survival, adhesion, proliferation, and thyroid hormone (T3 and T4) secretion of the thyroid cells when compared to a monolayer cell culture.

Other biomaterials which may be of interest include a vast range of hydrogels as they are soft and elastic, reflecting the intrinsic properties of native thyroid (163). Indeed, a study bioprinted a mouse thyroid construct within a collagen hydrogel (148). The endothelial cells derived from allantoic spheroids included in this construct established vascularization of the gland. Following engraftment below the kidney capsule, the construct was able to restore blood thyroxine levels and body temperature in athyroid mice. Another group utilized alginate–poly-L-ornithine-alginate to produce encapsulated thyroid follicles from porcine thyroid cells (158). This tri-layered scaffold significantly increased the level of T4 released from the follicles, indicating these may be applied to rescue a hypothyroid state.

### 3.2 ECM scaffolds

One of the main components of native tissue is the ECM which provides the microenvironment cells need to thrive. The majority of ECM, including the thyroid ECM, is composed of collagen, fibronectin, laminin and perlecan, forming collagen fibers and proteoglycan filaments, responsible for supplying tissue-reflective durability and tensile strength (166). As opposed to biomaterial scaffolds, the majority of ECM scaffolds are produced through the decellularization of native tissue, a process which utilizes detergents or mechanical manipulation to remove components that may elicit an inflammatory or immune response if implanted in a host (163). This causes the ECM to retain its biomechanical properties, composition, architecture, and biological activity, allowing it to direct cell migration and cell fate. The resulting decellularized ECM (dECM) scaffold can then be recellularized with the cell type of choice. Whilst the advantages of dECM scaffolds compared to biomaterial scaffolds include that it more closely mimics the required microenvironment, dECM scaffolds still have a potential for immunogenicity (167). Additionally, the tissue for these scaffolds needs to be obtained from healthy animals, proving to be less ethical than the production of some synthetic biomaterials.

There have been multiple attempts at producing a dECM scaffold for the regeneration of thyroid function. These most commonly utilize a decellularization process involving 1% sodium dodecyl sulfate (SDS), which is cytotoxic and requires extensive washing procedures as it is difficult to remove from the tissue due to its ionic nature (168). SDS is also known to cause alteration of the microstructure in some instances but no evidence of this was found in studies utilizing this decellularization approach on the thyroid (153, 157). Alternatively, one study by Strusi *et al.* used an approach combining trypsin, Triton-x100, peracetic acid, deoxycholic acid and ethylenediaminetetraacetic acid (EDTA) (156). Whilst this approach perfectly preserved the delicate stromal-vascular scaffold of the thyroid, peracetic acid and EDTA are known to increase the stiffness of the ECM and decrease the salt- and acid-soluble proteins (168). However, the mechanical properties and protein contents of the produced dECM scaffold were not assessed in this study.

A rat dECM scaffold which has been reseeded with the spontaneously immortalized rat thyroid cell line FRTL-5 has shown generation of three-dimensional structures resembling thyroid follicles *in vitro*, as well as synthesis of thyroglobulin and thyroid peroxidase, indicating thyroid-specific function (153). Whilst immortalized cell lines may not fully reflect the functionality of native tissue, these results have been confirmed in the same study by successful reseeding of the scaffold with primary human thyrocytes and maintenance of thyroglobulin and TPO gene expression. Additionally, another study involving the generation of a rabbit dECM scaffold reseeded with human thyrocytes indicated a functional model which did not induce cytotoxicity *in vitro* (157).

Instead, stem cells have been utilized to attempt the production of a functional dECM scaffold. One study focused on the recellularization of a stromal/vascular rat matrix with thyroid stem/precursor cells (155). Analysis of these indicated the formation of thyrocytes and heterotopic migration of the stem cells into the matrix septa where they orthotopically aggregated and gave rise to intracytoplasmic cavities, similar to those seen in native thyroid follicles. This scaffold secreted thyroid hormones for a minimum of seven days. Another study utilized thyroid islets derived from mesenchymal stem cells to recellularize a porcine liver organoid, which, upon implantation into mice, successfully rescued a hypothyroid state (149). Together these indicate that a stem cell approach can be used to produce a dECM scaffold which secretes thyroid hormone and can be used as treatment for hypothyroidism. However, it is noteworthy that only one of the dECM scaffolds has been tested *in vivo* (149).

Lastly, one study focused on the generation of an ECM scaffold without the use of decellularized tissue. This involved mincing of whole thyroid tissue, followed by digestions with collagenase and culturing on thermo-responsive Poly(N-isopropylacrylamide) (PIPAAm) dishes (147). Following culture of the cells, a slight change in the external temperature allowed controlled detachment of a thyroid cell sheet which, upon implantation in athyroid mice, significantly increased T3 and T4 serum levels.

## 4 Future outlook

Primary hypothyroidism is known to be more prevalent in females than in males, particularly during puberty and menopause (169). This has been partially attributed to an increased presence of estrogen in females which exhibits indirect effects on the thyroid economy, increasing thyroxine binding globulin, as discussed above (170). Additionally, excessive estrogen enhances inflammatory processes within the immune system, which increases the likelihood of autoimmune mediated thyroid damage occurring (171). These are factors which should be taken into consideration during the development of a scaffold. Estrogen also exhibits direct effects on thyroid cells, stimulating proliferation and migration (172). Through addition of this hormone to a bioengineered scaffold, this has potential to be exploited to enhance thyroid regeneration following gland damage.

Whilst the transplantation of primary thyroid cells presents as a reality in mice, the potential for immune attack on these cells remains, which would prevent this to be clinically applied as treatment for primary hypothyroidism. However, the discovery of iPSCs circumvents this issue. This technique allows any cell type to be reprogrammed into a pluripotent state through the induction of the genes Oct3/4, Sox2, c-myc and Klf4 (173). A vast number of studies describe the derivation of iPSCs from human tissue and as discussed above, these have been successfully differentiated into thyroid-like cells, able to produce thyroid hormones. This approach is most likely to be clinically beneficial as it negates the need to isolate thyroid cells from human tissue before damage occurs to the organ and would not require immunosuppressant therapy which would severely decrease quality of life of the patients.

It is not only the cellular component which may elicit an immunological response from the recipient; the materials used to fabricate these scaffolds also provide immunogenic potential and may lead to the development of an acute sterile inflammatory reaction termed a foreign body reaction (FBR). Scaffolds may induce a chronic neutrophil infiltrate, often leading to fibrosis around the site of implantation, decreasing the regenerative efficacy of the scaffold. However, immune responses are known to diverge between biopolymers and dECM scaffolds (174). Overall, synthetic biopolymers have a high potential for inducing an innate response whilst no risk is present for an adaptive response. Conversely, natural biopolymers present a low risk for both an innate and adaptive host immune response arising (175). Alternatively, clinically employed ECM material has been shown to prevent the formation of a fibrous capsule and instead induce a type-2-like immune response, through production of M2 macrophage and Th2 T cell, which has been correlated with an improved outcome of scaffold integration and tissue regeneration (174, 176, 177). However, many other scaffold properties including surface chemistry related to moiety and charge, and topography determine the immunomodulatory function of, hence immune response to the scaffold (178, 179). Furthermore, as with any invasive medical procedure a risk of infection is present. This, however, is reduced by the multiple injectable scaffolds currently under development, which make the scaffold implantation procedure minimally invasive (180, 181).

The functional activity of thyrocytes closely relates to their structural organization into follicles, with polarization being a key factor for hormone production. It was previously shown that monolayer culture of thyrocytes may cause loss of their functional activity (182). Hence, multiple three-dimensional *in vitro* approaches have been employed to maintain essential properties of thyroid tissue including cell polarization, intracellular contact, and presence of the intrafollicular lumens (183). The production of a scaffold which allows thyroid hormone secretion *in vivo* is therefore more likely to succeed if the material used allows the cells to assume the follicle confirmation required to achieve this. Taken together with the intrinsic elasticity of thyroid tissue, based on its relatively low young’s modulus, a softer material mirroring these properties may be most beneficial for this purpose (184).

Whilst the use of scaffolds composed of either decellularized tissue or natural polymers - although with the majority using Matrigel - has been extensively explored for the use of thyroid tissue engineering, this is not the case for synthetic polymers. Indeed, only one study has explored these by investigating the thyroid regenerative abilities of PCL or PLLA scaffolds (152). Synthetic polymers offer advantages in the form of tuneable properties and consistent, predictable mechanical-chemical properties (185, 186). However, while this indicates further exploration of their use in thyroid scaffolding may be of worth, utilized on their own they often display poor cell adhesion due to a lack of adhesion sites (187). The formulation of composite scaffolds would circumvent the issues associated with the use of either natural polymers, synthetic polymers or decellularized tissue as a single scaffold component. This may involve the incorporation of decellularized tissue into biopolymer scaffolds, which has been successfully used in other tissues and allows for recapitulation of the complex native ECM properties of the thyroid, adding increased cell viability, without compromising advantages of biopolymer scaffolds (188–190).

## 5 Conclusion

The application of bioengineering and regenerative medicine approaches to produce novel primary hypothyroidism therapeutics is promising. Investigations have been made into scaffolds fabricated using primary thyrocytes, but these prove difficult to culture. Additionally, a fully formed thyroid follicle is needed to produce thyroid hormones. Therefore, the utilization of stem cells may provide a better direction for further research, especially with the formation of thyroid organoids derived from these being well established. Both biopolymers and decellularized scaffolds have been used successfully to construct implants for the purpose of rescuing a hypothyroid phenotype. However, little diversity has been explored with regards to biopolymers, with the focus remaining on hydrogels, especially Matrigel. Future studies should address the usage of a wider variety of biopolymers, seeing as these have been applied with much success in other tissues. The recent advances in the application of composite scaffolds within tissue engineering also act as promising future avenues for thyroid applications. The amalgamation of various polymers with dECM should be further investigated as it has the possibility of providing a more favorable niche for cell growth due to the presence of native proteins, despite not losing any tuning potential. Lastly, only few of these constructed scaffolds have been investigated *in vivo*. Studies should be conducted regarding the implantation of these scaffolds *in vivo* as this will give us more insight into the physiological effects in a whole system. Whilst the scaffolding approach provides an auspicious outlook for primary hypothyroidism treatment, further work is required until clinical application.

## Author contributions

MH performed the literature review, drafted the manuscript and tables, and edited the manuscript. AC contributed to hypothesis generation and edited the manuscript. IN and EE edited the manuscript. All authors contributed to the article and approved the submitted version.

## Funding

This research was funded in whole by the Engineering and Physical Sciences Research Council (EPSRC) grant EP/T517884/1. This funding source had no role in the design and execution of this review or decision to submit results. For the purpose of open access the author has applied a creative commons attribution (CC BY) licence to any author accepted manuscript version arising. Additional funding was provided by the University of Edinburgh UKRI Open Access Fund.

## Conflict of interest

The authors declare that the research was conducted in the absence of any commercial or financial relationships that could be construed as a potential conflict of interest.

## Publisher’s note

All claims expressed in this article are solely those of the authors and do not necessarily represent those of their affiliated organizations, or those of the publisher, the editors and the reviewers. Any product that may be evaluated in this article, or claim that may be made by its manufacturer, is not guaranteed or endorsed by the publisher.
